# Twin Pregnancy With Discordant Pathology: A Case of Molar Gestation and Viable Co-twin After Assisted Conception

**DOI:** 10.7759/cureus.93266

**Published:** 2025-09-26

**Authors:** Acha B Adam, Najla S Gubari

**Affiliations:** 1 Obstetrics and Gynecology, Aya Specialist Hospital, Jeddah, SAU

**Keywords:** assisted reproductive technology, coexisting viable twin, complete hydatidiform mole, gestational trophoblastic disease, in vitro fertilization, pregnancy termination, subchorionic hematoma, twin pregnancy, ultrasound, β-hcg

## Abstract

Complete hydatidiform mole with a coexisting viable twin is an exceptionally rare condition. It poses significant maternal risks, including hemorrhage, early-onset preeclampsia, and progression to gestational trophoblastic neoplasia. The use of assisted reproductive technology (ART) has increased the detection of such complex gestations, yet optimal management remains controversial. We present the case of a 23-year-old primigravida who conceived a dichorionic diamniotic twin pregnancy following frozen embryo transfer. Early ultrasound demonstrated one normally developing gestational sac with a viable fetus and a second irregular, avascular sac with cystic degeneration. Serum β-human chorionic gonadotropin (β-hCG) levels were markedly elevated for gestational age. Over the following weeks, the abnormal sac exhibited progressive molar changes, accompanied by recurrent vaginal bleeding and a persistent subchorionic hematoma. The viable co-twin continued to grow appropriately. Given the escalating maternal risk, multidisciplinary counseling was undertaken, and the patient elected to terminate the pregnancy. Suction evacuation was performed without complication. Histopathological examination confirmed hydatidiform mole. Post-evacuation β-hCG levels declined rapidly, and the patient remained well on serial follow-up. This case illustrates the diagnostic and management challenges of a complete hydatidiform mole with a coexisting viable twin in an ART pregnancy. Early diagnosis, close surveillance, and multidisciplinary counseling are critical for balancing maternal safety with fetal viability in these high-risk gestations.

## Introduction

Twin pregnancy with a coexisting molar gestation is a rare and challenging clinical entity, occurring in approximately 1 in 22,000-100,000 pregnancies [1,2]. It is most often associated with a complete hydatidiform mole alongside a viable fetus [3]. The condition is characterized by a markedly elevated serum β-human chorionic gonadotropin (β-hCG) level, distinctive ultrasonographic features, and a substantial risk of maternal morbidity, including severe hemorrhage, early-onset preeclampsia, hyperthyroidism, and progression to gestational trophoblastic neoplasia [2,4].

The introduction of assisted reproductive technologies (ARTs), particularly in vitro fertilization (IVF), has led to an increase in multiple gestations and, in turn, a higher recognition of rare combinations of gestational pathologies. The pathogenesis of molar pregnancy in a twin gestation following ART remains unclear [3].

The clinical course of these pregnancies is variable. Some may progress to late gestation with the delivery of a healthy co-twin, whereas others require termination because of maternal compromise or evidence of malignant transformation [4]. Management is further complicated by the emotional and ethical implications of terminating a pregnancy with a viable fetus. These decisions necessitate a multidisciplinary approach involving maternal-fetal medicine specialists, oncologists, and neonatologists [1,5].

This case describes a rare occurrence of a dichorionic diamniotic twin pregnancy conceived via IVF, in which one gestational sac demonstrated features of a complete hydatidiform mole and the other contained a viable fetus. The report highlights the diagnostic challenges, potential maternal risks, and the importance of individualized counseling in formulating an appropriate management plan.

## Case presentation

A 23-year-old primigravida with a history of unexplained primary infertility conceived a dichorionic diamniotic twin pregnancy following frozen embryo transfer of two high-quality blastocysts. Her medical and surgical history was otherwise unremarkable, and she had no known drug allergies. She was receiving luteal support with vaginal progesterone (cyclogest 400 mg twice daily) and intramuscular hydroxyprogesterone caproate (500 mg every 72 hours).

At approximately 8 weeks’ gestation, she presented to the emergency department with recurrent episodes of vaginal bleeding accompanied by clots. Physical examination revealed stable vital signs and a closed cervix. Serum β-hCG was markedly elevated. Transvaginal ultrasonography demonstrated a bulky gravid uterus with two gestational sacs: one irregular and distorted sac without a fetal pole, and a second regular, fundally located sac containing a viable embryo (crown-rump length (CRL) consistent with 8 weeks + 3 days; fetal heartrate (FHR) 179 beats/minute).

Over the following three weeks, she experienced repeated episodes of vaginal bleeding requiring multiple hospital visits. Serial ultrasound examinations consistently showed ongoing viability of one fetus, accompanied by a persistent subchorionic hematoma adjacent to the abnormal sac. The viable sac demonstrated appropriate interval growth and cardiac activity. The abnormal sac progressively developed features of molar degeneration, including irregular contour, semi-turbid echogenic contents, diffuse cystic changes, and absence of vascularity on Doppler imaging.

**Figure 1:**
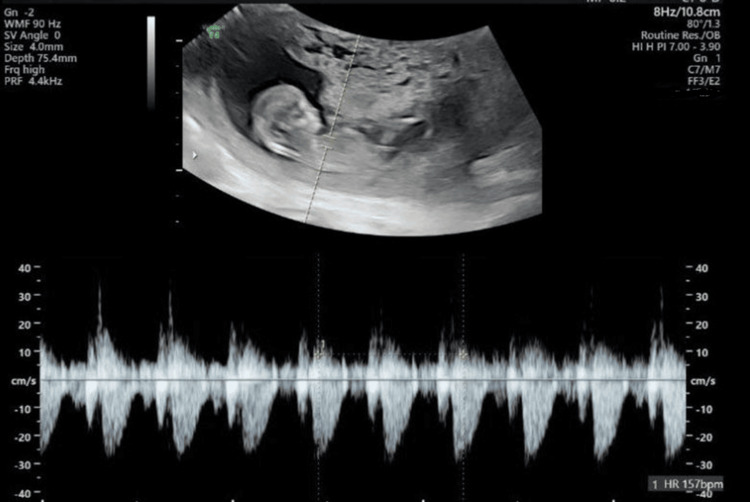
Ultrasound showing the viable co-twin (Twin A) with an appropriate crown–rump length and normal nuchal translucency at the end of the first trimester.

**Figure 2:**
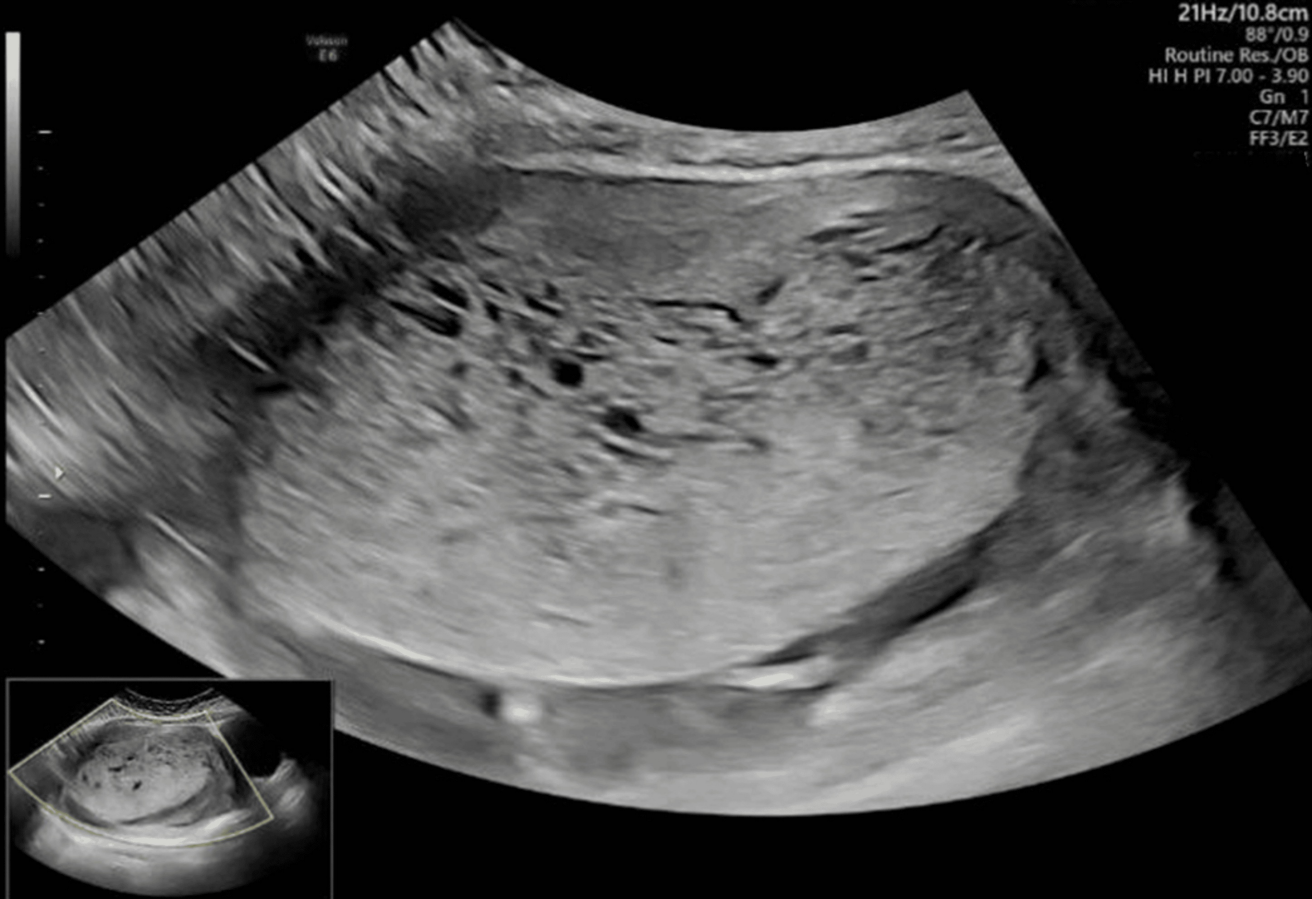
Ultrasound image demonstrating the abnormal gestational sac with features of complete molar degeneration, including diffuse cystic changes and absence of vascularity on Doppler imaging.

By the end of the first trimester, detailed sonography confirmed the presence of a viable fetus with normal nuchal translucency and appropriate growth parameters (Figure 1), along with a second abnormal sac measuring several centimeters in diameter, avascular, and consistent with complete molar changes (Figure 2). Serum β-hCG had risen to markedly supraphysiologic levels for gestational age.

Given the high risk of significant maternal complications, including hemorrhage, preeclampsia, and potential progression to gestational trophoblastic disease, the patient underwent multidisciplinary counseling. After careful discussion of the risks and benefits, she opted for the termination of pregnancy. Suction evacuation of both gestational sacs was performed without intraoperative complications. Histopathological examination confirmed hydatidiform mole, characterized by enlarged hydropic villi and focal trophoblastic hyperplasia. Post-evacuation, serum β-hCG levels demonstrated a rapid and sustained decline on serial weekly measurements, and the patient was scheduled for continued monitoring until complete normalization.

## Discussion

This case of a complete hydatidiform mole coexisting with a viable twin in an IVF-conceived dichorionic diamniotic pregnancy underscores several important clinical and ethical considerations. Co-twin molar gestations are rare, and complete moles even more so; fewer than 100 cases have been reported in the literature, with only a handful associated with assisted reproductive technologies [3]. The pathogenesis likely occurs when an empty ovum is fertilized by either one sperm that duplicates its DNA, resulting in a diploid 46, XX karyotype, or by two different sperms, resulting in a diploid 46, XY karyotype [6].

Clinically, the distinguishing ultrasonographic features - an irregular, cystic gestational sac lacking vascular flow juxtaposed to a normally developing sac - are critical for early diagnosis. Markedly elevated β-hCG levels beyond expected ranges for gestational age may further raise suspicion [3]. However, the presence of a viable fetus can create diagnostic uncertainty, and serial imaging combined with quantitative β-hCG monitoring is essential to delineate the divergent courses of the two sacs. Subchorionic hematoma formation, as observed in this patient, is a common complication that can exacerbate bleeding and may mimic impending miscarriage [4,7].

Management of twin molar pregnancies must balance maternal safety against the potential for a healthy live birth. Reported outcomes vary: live birth rates approach 40%-50% when selectively continuing the co-twin, but maternal risks, including massive hemorrhage, early-onset preeclampsia, hyperthyroidism, and progression to gestational trophoblastic neoplasia, are substantial [4]. In ART pregnancies, emotional investment and expectations may further complicate decision-making. A multidisciplinary team comprising maternal-fetal medicine specialists, oncologists, and neonatologists was indispensable for counseling, surveillance, and intervention planning.

Termination is generally recommended when maternal complications arise or when molar degeneration progresses, as occurred here. Suction evacuation with careful follow-up of β-hCG levels remains the standard of care to minimize the risk of persistent trophoblastic disease [4]. Histopathology confirming molar changes guides surveillance intensity. In this patient, rapid post-evacuation decline in β-hCG and absence of metastatic disease on imaging allowed for conservative follow-up without chemotherapy.

This case highlights the need for vigilance in multiple gestations conceived via IVF, especially when abnormal sonographic findings or disproportionate β-hCG elevations occur. Early recognition, transparent counseling, and a coordinated multidisciplinary approach are paramount in managing these complex pregnancies to optimize maternal outcomes while respecting patient autonomy.

## Conclusions

Complete hydatidiform mole with a coexisting viable twin is an exceptionally rare and high-risk pregnancy, particularly in the setting of assisted reproductive technology. Early diagnosis through meticulous ultrasonographic assessment and β-hCG monitoring is crucial, as timely recognition enables informed counseling and individualized management. Given the potential for life-threatening maternal complications and malignant transformation, these cases require a multidisciplinary approach that prioritizes maternal safety while acknowledging the complex emotional and ethical dimensions of decision-making. This case underscores the importance of balancing maternal and fetal considerations in rare twin gestations with discordant pathology.
